# Human malarial disease: a consequence of inflammatory cytokine release

**DOI:** 10.1186/1475-2875-5-85

**Published:** 2006-10-10

**Authors:** Ian A Clark, Alison C Budd, Lisa M Alleva, William B Cowden

**Affiliations:** 1 School of Biochemistry and Molecular Biology, Australian National University, Canberra, ACT 0200, Australia; 2 John Curtin School of Medical Research, Australian National University, Canberra, ACT 0200, Australia

## Abstract

Malaria causes an acute systemic human disease that bears many similarities, both clinically and mechanistically, to those caused by bacteria, rickettsia, and viruses. Over the past few decades, a literature has emerged that argues for most of the pathology seen in all of these infectious diseases being explained by activation of the inflammatory system, with the balance between the pro and anti-inflammatory cytokines being tipped towards the onset of systemic inflammation. Although not often expressed in energy terms, there is, when reduced to biochemical essentials, wide agreement that infection with falciparum malaria is often fatal because mitochondria are unable to generate enough ATP to maintain normal cellular function. Most, however, would contend that this largely occurs because sequestered parasitized red cells prevent sufficient oxygen getting to where it is needed. This review considers the evidence that an equally or more important way ATP deficency arises in malaria, as well as these other infectious diseases, is an inability of mitochondria, through the effects of inflammatory cytokines on their function, to utilise available oxygen. This activity of these cytokines, plus their capacity to control the pathways through which oxygen supply to mitochondria are restricted (particularly through directing sequestration and driving anaemia), combine to make falciparum malaria primarily an inflammatory cytokine-driven disease.

## Background

The mechanism of the disease caused by *Plasmodium falciparum*, arguably the pathogen that causes the most human suffering, has been hotly debated for many decades. Clearly, rational adjunct therapy depends on getting this right. For over twenty years the central debate has come from two apparently opposing camps. One champions the mechanical hypothesis, based on the concept of insufficient oxygen reaching vital organs, and the other the cytokine hypothesis, in which excessive release of pro-inflammatory cytokines are the primary driving force of disease and death. The former concept stresses the uniqueness of the pathophysiology of falciparum malaria compared to that of other severe systemic infectious diseases, whereas the latter sees malaria as having fundamentally the same basis as these other conditions, with the adhesive property of parasitized erythrocytes giving it no more than a distinctive flavour.

Critical analysis of the mechanism of falciparum malarial disease would not have been possible without the seminal work of Peter Mitchell [1,2], who identified mitochondria as the ATP-generating powerhouse of aerobic cells, and thus of all aerobic organisms. Among the doors this opened was the opportunity to understand severe infectious disease by seeing it through the perspective of these organelles. This review largely discusses the relative contribution to disease processes of mitochondria being prevented from getting enough oxygen, and being able to use all the oxygen that reaches them, and the combined effects of these two influences. This includes blood flow restriction in microvessels by parasitized red cells that adhere to endothelial walls (sequestration), the potential restriction to oxygen supply that is unique to falciparum malaria. As discussed below, the broader literature is consistent with the view that sequestration during severe disease is not a passive event that is simply an amplification of what occurs early in infection and in tolerant individuals, but one in which location and avidity of adherence, and, therefore, pathogenic effects, are controlled by inflammatory cytokines.

Despite advances in understanding diseases clinically very similar to falciparum malaria in terms of inflammatory cytokines, enthusiasm for the mechanical obstruction hypothesis seems at least as strong as ever [3-5]. Although much literature now demonstrates dependence of the mechanisms of poor oxygen delivery on excess inflammatory cytokine release, even the most recent members of the vaso-occlusion school [5] still see the cytokine theory of disease as an alternative to be argued against rather than as an essential component of their own disease model. Hence it is timely to bring together an update of the evidence why cytokines are regarded as so central to this disease. In particular, it seems warranted to summarize how broadly the harmful influences of the inflammatory cytokines are now known to extend. By introducing concepts in infectious disease in general, and then moving to the particular case of falciparum malaria, this review expands on these functionally interconnected consequences of excess production of inflammatory cytokines.

## Systemic infectious diseases and inflammatory cytokines

There is now remarkably widespread acceptance that cytokines such as TNF and interleukin-1 (see "cytokine storm" in Google) are the essential mechanism of systemic disease caused by infectious agents. Indeed, one would be hard pressed to find an alternative explanation now current for the anorexia, tiredness, aching joints and muscles, fever and sleepiness that patients experience in any systemic infection, including both vivax and falciparum malaria. Neither is it disputed that exacerbated release of these same mediators is the best current line of investigation for the mechanism of severe and life-threatening illness, such as sepsis [6] and influenza [7]. The difficulty, confined to falciparum malaria, is to get its broad acceptance in a research community that has traditionally seen its disease as unique, mechanistically separated from other infectious conditions by the presence of sequestered parasitized red cells often seen in certain intravascular locations at autopsy. To some researchers sequestered parasites are still necessary and sufficient for illness from falciparum malaria to occur, and to cause fatality [4,8]. Much of this section will include parallels between malaria and similar diseases caused by other pathogens, and basic research done on the effects of inflammatory cytokines on normal cells. It also recounts, for the present-day audience, the malarial origin of this concept of disease pathogenesis.

### Intra-erythrocytic death of haemoprotozoa, the original link of TNF to disease

Nearly thirty years ago, ignorance of accepted malaria wisdom, in a London tumour/virology lab where this attribute was universal, allowed a novel tumour-oriented interpretation to be put on the observation that the non-lethal mouse parasite, *Babesia microti*, was killed by the immune system in circulating red cells [9]. This also happened in some species of malaria parasites with which it cross-protected. Encouragement that this phenomenon was worth pursuing came from the Guy's Hospital group, then at the top of the malarial immunity tree, who observed the same unexpected and puzzling phenomenon when they challenged malaria-immunized rhesus monkeys [10].

Of particular concern for the then official Guy's dogma (malarial immunity operates through a specific antibody focussed on the merozoite surface, adopted unquestioned by other major vaccine groups), even unrelated parasites died *en masse *inside red cells when previously immunized monkeys were challenged [11]. In their view this could offer an explanation of why some monkeys had high levels of antibody expected to be protective, yet failed to resist a challenge infection [11], while others with little or no anti-merozoite antibody were immune [12]. A report from the US told a similar tale [13]. Clearly, some powerful influence beyond specific antibody, and inconvenient for mainstream thinking on immunity and vaccine development, was reproducibly occurring.

### Tumour necrosis factor (TNF), the prototype inflammatory cytokine

The observation that pre-treatment with the Bacillus Calmette-Guérin (BCG) strain of *Mycobacterium tuberculosis *controlled a subsequent infection with any of several strains of babesia or malaria in mice (no antibody, parasites dying in red cells, not phagocytes) was fortuitously timed with the publication of the first paper on TNF [14]. This allowed us, in collaboration with these New York tumour researchers, to propose novel roles for TNF in immunity and disease pathogenesis in malaria and sepsis [15,16]. In summary, through linking the protective capacity of agents such as BCG with the degree to which they sensitised to bacterial lipopolysaccharide (LPS) it came to be realised that the pathology of LPS toxicity (and subsequently that of rTNF) and *Plasmodium vinckei *infections in mice were largely identical, cytokine-mediated, events. As noted in a contribution to Brian Maegraith's 1988 Festschrift Proceedings [17], the cytokine approach gave teeth to his inflammation-based arguments on malaria disease of forty years earlier [18].

As reviewed in 1987 [19], this experience with mouse babesiosis and malaria provided the insight that this anti-tumour mediator arguably had roles in both cell-mediated immunity (CMI) and the pathogenesis of infectious disease in general. As well as malaria, this concept was reasoned in 1981 to explain the mechanism of typhoid [15], sepsis in general [16], and viral diseases in 1989 [20], and it eventually spread across all acute infectious diseases (see [21] for a recent review). For example, within a few years it began to dominate the sepsis literature [22,23], and the virulence of different strains of influenza, a disease that is a standard clinical misdiagnosis for imported malaria, has now been expressed in terms of their capacity to induce TNF [24]. While still engendering strong opposition from some malaria researchers [4,25], these ideas have been readily accepted by scientists working on bacterial, rickettsial or viral diseases. A broad literature across infectious disease now describes inflammatory cytokines as having a beneficial role in host defence, but being harmful to the host if produced excessively. Indeed, the acceptance and applicability of this concept is now general enough for its biological evolution to be an independent subject for research [26].

Once neutralizing anti-TNF antibodies became available for human use, they were tested by others for efficacy against malarial parasites and disease. Unfortunately a central tenet of the concept (that the pro-inflammatory cytokines that cause disease are the same mediators that, in lower concentrations, are responsible for the innate immunity that controls parasite growth – see also tuberculosis etc. in next paragraph) was not adequately considered. TNF has been shown to inhibit a mouse malaria parasite *in vivo *[27], and *Plasmodium falciparum in vitro*, provided white cells to generate the next down-stream mediator, possibly nitric oxide [28], were present [29]. This is consistent with findings in human subjects [30]. Thus it is not surprising that anti-TNF antibody, by removing inhibitory pressure from the pathogen, can enhance the disease in falciparum malaria [31], as shown five years earlier in human sepsis [32].

The broad relevance of these malaria-origin concepts in immunity and disease is best illustrated by noting the consequence of passive vaccination against TNF for Crohn's disease and rheumatoid arthritis, now a large-scale routine treatment [33]. Its practical success puts the relevance of these pro-inflammatory cytokines in human inflammatory disease beyond doubt, and the major side effect (pre-existing or acquired tuberculosis, salmonellosis, or listeriosis becoming fulminant) nicely demonstrates its relevance to CMI against many pathogens. It is unlikely to be coincidental that all three that flare are on our list of organisms that protected against haemoprotozoan parasites, causing intra-erythrocytic death, and priming mice for TNF production [34,35]. Evidently the host was setting up a cell-mediated response that would protect against these organisms. Being non-specific in nature, it also protected against haemoprotozoa as well. From this reasoning *Coxiella burnetii*, a crude extract of which was an extremely good protectant [36], and primer for TNF (E. Carswell, pers. comm.) will also predictably flare if anti-TNF is given, long term, to an arthritis patient harbouring this human pathogen.

### The inflammatory cytokines as a group

In this text, TNF is used as a term of convenience to designate the pro-inflammatory cytokines as a whole. Other cytokines, such as lymphotoxin (LT), interleukin-1 (IL-1), interleukin-6 (IL-6) and soluble Fas ligand (FasL) serve similar functions. In passing, it warrants noting that the term TNF-alpha, while still common, has been obsolete ever since LT ceased being referred to as TNF-beta and reverted to its original name, allowing TNF to do the same. Although the literature connecting the pro-inflammatory cytokines other than TNF to malaria [37-40] is as yet much smaller than that for TNF, this does not imply that their potential for understanding this disease is correspondingly minute. A TNF superfamily of 19 members signalling through 29 receptors has more recently been described [41]. Many of these mediators induce other cytokines and enzymes that add to the inflammatory cascade. For example, TNF induces migration inhibitory factor (MIF) [42,43], and TNF, IL-1 beta and LT generate the inducible form of nitric oxide synthase iNOS [44]. Anti-inflammatory cytokines such as IL-10, IL-4, and transforming growth factor-beta (TGF-beta), also play active roles, and an imbalance between these and their pro-inflammatory counterparts often determines outcome in disease. Some tens of thousands of publications on inflammatory cytokines and systemic inflammatory and other disease now exist.

The pro-inflammatory cytokines most closely investigated in malaria, such as TNF, usually act as homeostatic agents, but can cause pathology if produced excessively. A recently defined example is MIF (see above paragraph), belonging to an ancient gene family, with structural homologues in bacterial organisms. As an indication of the broad relevance and complexity of these cytokines and their interactions, MIF is also induced in mammals by *E. coli *lipopolysaccharide [42], staphylococcus toxic shock toxin, and streptococcal pyrogenic toxin A [45]. Whether this is direct, or via the TNF they induce, appears not to have been ascertained. Likewise, MIF can act directly, through TNF, or in synergy with it, in generating anaemia, as discussed below. It is, as noted above, TNF-induced, and remarkable for several reasons, one being that its description 40 years ago [46,47], began the concept of what are now called cytokines. Ten years later, and long before MIF was realised to have functions other than migration inhibition and pathogenicity in sepsis (which its inhibition suppresses, in a realistic model, most impressively [48]), it was the first cytokine described in a malaria infection [49]. A few years after its rediscovery as a homeostatic glucocorticoid antagonist [50,51], it has become central to understanding malarial anaemia, as discussed below. It is also increased in malarial placentas [52].

Cytokines such as TNF and IL-1, both increased in a wide range of systemic inflammatory diseases, including falciparum malaria, can induce a late-onset, but long-acting wave of a cytokine termed the high mobility group box 1 (HMGB1) protein, which prolongs and amplifies inflammation [53,54]. This molecule, previously known for several physiological functions, now shows great promise as a therapeutic target in sepsis, in that countering it after the onset of illness has been reported to protect well in experimental sepsis [55,56]. HMGB1 has been shown to be increased, in proportion to degree of illness, in serum from African children infected with falciparum malaria [57]. Like TNF, HMGB1 has roles in other inflammatory diseases [58], reaffirming malaria's position within their ranks. The malarial context of HMGB1 is reviewed more fully elsewhere [21].

### TNF, a tool to determine the nature of malarial toxin

The idea of malarial disease being caused by parasites releasing a toxin is even more venerable than that of vaso-occlusion, since it is based on a report by Golgi in 1886 [59], in which he noted onset of fever and rigors at a predictable short interval after the regular shower of new parasites escape from bursting red cells. These principles were much discussed in the first decade of the 20th century [60]. Clearly, something like this was needed to explain how tissue not invaded by the parasite was nevertheless damaged during falciparum malaria. Examples are sites such as the adult kidney and lung, where dysfunction can be catastrophic, yet sequestration never obvious, and often absent. The toxin idea lay fallow for many decades, not helped, in hindsight, by the underlying assumption that toxicity arose directly from a parasite product, in the manner of tetanus toxin.

The proposal that malarial products were not harmful in themselves, but only through causing the infected host to harm itself through generating toxic amounts of molecules that, in lower concentrations, inhibit growth of malarial parasites, gave the toxin concept new impetus [15,16,19]. These papers predicted that the nature of the malarial product that triggers illness could be defined through its ability to induce release of TNF from mammalian cells. A group in London did much work along these lines in the late 1980s and early 1990s [61,62], and concluded it was closely related to phosphotidylinositol (PI) [63]. Others extended this argument to the glycosylated form of this molecule (GPI) [64]. The original proposal that malarial toxin operates through inducing generation of TNF and related cytokines was greatly strengthened when immunizing mice against GPI and then infecting them with one of the mouse malaria parasites protected against certain pathology that TNF causes on injection [65]. Indeed, this study reports having established that GPI appears sufficient and necessary for the induction by malarial parasites of host pro-inflammatory responses *in vitro*. The field has been well reviewed recently [66], with these authors and others expressing doubts about the wisdom of vaccinating against GPI to prevent malarial disease [21,66,67]. As noted some years ago, the need for sufficient TNF to allow immune activation to proceed normally during infections is plausibly why this potentially lethal mediator has survived 300–500 million years of evolution [68]. However, despite recent reaffirmation of the GPI/cytokine/disease concept [69], the group that first suggested that GPI was the main TNF inducer in malaria appear to have recently [25] changed their disease model to one that eliminates a requirement for inflammatory cytokines. In view of GPI having been identified through its capacity to induce pro-inflammatory cytokines, it would have been remarkable to chance upon a molecule that induces these mediators, yet mimicks their actions in their absence. Clarification awaits a more detailed report.

### Breadth, and acceptance, of the cytokine concept of disease pathogenesis

The extensive parallels that exist between the sepsis and malaria literature can be viewed from the perspective of the wide range of functionally-important inflammatory cytokines present in the circulation in both conditions (Table 1). This strengthens the view that the two diseases operate through very similar mechanisms. Nevertheless, a group working with African children has recently advocated that in order to understand falciparum malaria disease one must return to the pre-cytokine era. They evidently still espouse the idea that local vaso-occlusion uniquely sets the organ pathology of this disease apart from others with which it is clinically confusable, in particular sepsis [3-5,70]. Since the failure of treatment with corticosteroids to ameliorate severe cerebral malaria has been used as evidence against cytokine involvement [4], it warrants recalling that MIF, known to be high in this circumstance [71], antagonizes glucocorticoids [72], and nitric oxide (noting iNOS is also high [71]) inhibits glucocorticoid binding to its receptor [73]. Moreover, this data rationalizes the failure of corticosteroid as a treatment.

**Table 1:** Some changes common to systemic inflammatory states in general, including sepsis and falciparum malaria

TNF, IL-1, iNOS and IFN-gamma, MIF, IL-10 and HO-1 raised
gamma-delta T cells raised
MRP8 (S100A8) and MRP14 (S100A9) raised
Procalcitonin raised
HMBG1 raised
ICAM, VCAM and p-selectin raised
insulin resistance
hyperlactataemia
hypoglycaemia
metabolic acidosis
hyponatraemia
coagulopathy
thrombocytopaenia
decreased red cell deformability

Analogy with other diseases is still an under-exploited tool in malaria. The original interest in TNF as a possible mediator of both innate immunity and disease pathogenesis in infectious disease came from analogies between the ability of BCG to protect against both tumours and intra-erythrocytic protozoa [9]. When introducing the excess inflammatory cytokine concept in 1981 [15], a common case for malaria, gram-negative bacteria and the Jarisch-Herxheimer reaction, all of which withstood the test of time, was argued by analogy. As reviewed recently [21], the range of infectious diseases that come under the systemic inflammation umbrella now extends beyond bacterial diseases to those caused by rickettsias, protozoa other than malaria, and viruses. Moreover, increased circulating levels of TNF and functionally similar cytokines have been measured in the serum very soon after onset of illness in virtually all those infectious diseases in which it has been sought. In addition, essentially all of the signs and symptoms involved in the clinical confusability of malaria and other causes of fever were inadvertently reproduced during the era when rTNF was being injected in volunteers as an antitumour agent [74,75]. This includes headache, fever and rigors, nausea and vomiting, diarrhoea, anorexia, myalgia, thrombocytopaenia, immunosuppression, coagulopathy and central nervous system manifestations, all of which have a literature on a mechanism through inflammatory cytokines. The rate, timing and intensity of cytokine (pro- as well as anti-inflammatory) release will vary in different disease states, and also between individuals, and provide them with somewhat distinctive clinical pictures, but the fundamentals remain. The clinical patterns generated are remarkably close, in that, at least in some populations, clinical features cannot predict a diagnosis of malaria from other causes of fever [76].

The principle extends beyond infectious diseases. A number of non-infectious states fit this pattern, with excessive release of pro-inflammatory cytokines producing a systemic inflammatory response. As in malaria and sepsis, metabolic acidosis [77,78], hyperlactataemia [79,80] and encephalopathy are seen in tissue injury syndromes such as heatstroke, trauma, and burns. As reviewed [21], all of these conditions are ripe for an explanation in terms of HMGB1, liberated from the nuclei of damaged tissue [81], setting the scene for a broad range of inflammatory cytokine release. Iatrogenic cytokine release syndromes, such as the side effects of OKT3 therapy [82], and acute graft versus host disease reaction [83,84] can also exhibit these changes, including a reversible encephalopathy. In both of these conditions the relevant pro-inflammatory cytokines are produced excessively, and where tested (side effects of OKT3 therapy [85], and acute graft-versus-host disease reaction [86]), prior exposure to neutralizing antibody directed against TNF prevents illness.

As with much research on neutralizing antibody to TNF, this outcome does not imply that TNF is more important than, for example, IL-1 in this context, since anti-IL-1 antibodies have rarely been tried. Blocking IL-1, and indeed IL-1 and TNF simultaneously, are in their infancy, but show promise [87,88]. Likewise, research into the disease aspects of LT, present in falciparum malaria, and relevant to the mouse model of cerebral malaria, is relatively ignored, largely through difficulty of obtaining reagents. Additional strong evidence for inflammatory cytokines and falciparum malaria being functionally intertwined comes from studies on variation in the human genome in Africa [89-91]. It is now accepted that falciparum malaria, historically the major fatal endemic disease in much of this continent, is associated with polymorphisms of these pro-inflammatory cytokines and iNOS, which are induced in this disease. Not surprisingly, sepsis and meningococcal disease have a similar literature [92,93]. Like any other DNA trying to survive, that of humans uses trial and error to adapt itself to its surroundings, leaving a trail of evidence as it does so.

In summary, illnesses arising from excessive systemic production of inflammatory cytokines include not just malaria and sepsis, but many more infectious, and non-infectious, diseases. Insights gained by recognizing the value of argument by analogy across this wide spectrum have been immense, and the general concept is now so firmly entrenched that, as noted earlier, its influence on the evolutionary effects of infectious disease is a research topic in its own right [26]. It would, therefore, be most unexpected were the illness of falciparum malaria, so clinically confusable with other infectious diseases, and known to generate the same inflammatory cytokines as they do, to arise from an unrelated mechanism [5,25].

## Mitochondria unable to use available oxygen, a primary effect of inflammation

### Inflammatory cytokines reduce ability of mitochondria to use oxygen

One of the many actions of the cytokines responsible for systemic inflammation is to disable oxidative phosphorylation within mitochondria. This is reflected in the hyperlactataemia commonly seen in severe infectious disease, and correlating with outcome. This is not to suggest that oxygen supply and its utilization are not often limited simultaneously, and interact. Indeed, if toxin is replaced by its downstream consequence (the effects of pro-inflammatory cytokines) such interaction was proposed for malaria by Meleney over 60 years ago [94].

Both sepsis and malaria researchers have shown that injecting TNF, the prototype inflammatory cytokine, increased in both diseases, causes hyperlactataemia [95,96], and blood lactate levels in severe malaria have proved to correlate closely with levels of both TNF and interleukin-1 [97]. Nevertheless, it is fair to say that sepsis researchers, without the tradition of a primary role for sequestration to defend, have been more receptive than malariologists to the systemic inflammatory explanation for altered carbohydrate metabolism, and more readily pursued it. Thus, they were more open than most of their malaria counterparts for the insights of the early 1990s, when newer techniques demonstrated that oxygen tension in tissues was actually increased (not decreased, as the literature predicted) in septic rats [98], patients [99], and pigs [100]. This implied an inability of mitochondria to utilize oxygen, forcing glycolysis to compensate, as best it can, for the energy deficit. The next insights came from groups who developed the cytokine-induced mitochondrial dysfunction model of disease [101-103] and, thus, provided an inflammation-based explanation for a shutdown of aerobic glycolysis, a consequent increased rate of glycolysis, and thus lactate production, metabolic acidosis and cellular energy depletion. In any disease with high levels of inflammatory cytokines this mimics poor oxygen supply. An important difference, however, is that the effects of the nitric oxide through which mitochondrial shutdown largely operates are reversible [104,105], whereas frank hypoxia, through vaso-occlusion, as evidenced by the stroke literature, is less so. Thus, the former, not the latter, is consistent with the marked reversibility of metabolic comas [106], a term advocated [21,107] to include human malaria.

This approach to understanding energy balance in sepsis has been followed successfully on a number of cell types, including hindlimb skeletal myocytes, gut wall cells, and hepatocytes. The wide range of tissues in which these concepts have been demonstrated adds to the arguments on systemic origins of lactate in sepsis and malaria. For example, inflammatory cytokines have been shown to cause contractile dysfunction [108,109] and also energy depletion [110,111] through effects, often mediated through induced nitric oxide [109,112], on cardiac muscle. Likewise, in diaphragmatic skeletal muscle there is evidence of cytokine-induced nitric oxide [113,114] and oxygen-derived free radicals [115] combining to form peroxynitrite [116,117] and this causing dysfunction of mitochondria in myocytes, leading to energy depletion and thus muscular contractile failure. The outcome here is to reduce the patient's ability to counter acidosis by blowing off CO_2_.

An additional pathway through which the inflammatory cytokines may reduce oxygen consumption is through peroxynitrate (OONO^-^), a product of NO (from iNOS induced by these cytokines) and superoxide, overactivating poly(ADP ribose) polymerase-1 (PARP-1) [118]. This can deplete cellular stores of NAD^+^, and efforts to resynthesise it can deplete ATP as well (reviewed in reference [119]). Moreover, NAD^+ ^is essential for glycolysis, so its depletion can be expected to impair glycolytic input into mitochondria. These concepts were reviewed in depth in a malaria context a few years ago [107].

## Mitochondria starved of oxygen, a secondary effect of inflammation

This section summarizes the ways in which inflammatory cytokines indirectly limit the supply of oxygen to cells, and thus further reduce the capacity of their mitochondria to generate ATP through oxidative phosphorylation. There are good arguments from the basic literature that they may do so through directing sequestration towards organs that are particularly oxygen-sensitive. Being a new concept in the malarial world, this literature is examined here in some detail. In addition, anaemia, cardiac insufficiency, or insufficient circulating volume (see below, and Figure 3) can all be driven by inflammatory cytokines. Again, infectious disease in general is outlined before focussing on the particular case of falciparum malaria.

### Inflammatory cytokines cause blood elements to adhere to endothelium

It is well accepted that upregulation by inflammatory cytokines of adhesion sites on endothelial cells invites susceptible circulating blood elements to attach to the inner wall of blood vessels. In many diseases, including malaria, this includes activated leukocytes and platelets, both of which play important roles in promoting procoagulant activity. For example, in malaria this activity is seen on circulating monocytes [120] and placental macrophages [121], and the thrombin so formed enhances adhesion by increasing expression of CD36 on platelet surfaces [122].

#### Platelets and leukocytes

Platelets and leukocytes have been reported to adhere to endothelium in viral [123-125], bacterial [126,127] and protozoal infections, including the cerebral vasculature in paediatric falciparum malaria [71,128]. In particular, aggregated monocytes are a striking feature in malarial placentas [129], and a less dramatic finding in cerebral vessels [130], where they and the nearby endothelial cells stain strongly for iNOS [71]. These adhering elements can set up local foci of inflammation, generating more inflammatory cytokines (eg TNF in placentas [131]), including inflammatory cascades initiated by HMGB1 released from the adhering activated platelets [132]. Since HMGB1 increases are associated with severity of falciparum malaria [57], this could account for the potentiating effects of platelets reported in an *in vitro *model of endothelial activation by *P. falciparum *[133]. Along with the effects of systemic inflammation, these local inflammatory foci contribute to potentially fatal pathology, including loss of endothelial integrity, in infectious diseases in which circulating inflammatory cytokine levels are sufficiently increased. Notably, the range of complications seen in falciparum malaria, including coma, can therefore develop in sepsis [134], influenza [135], and sometimes vivax malaria [136,137], which show evidence of a high systemic inflammatory response, but have no possible involvement of sequestering parasites.

#### Parasitized red cells, an additional circulating adherent element in falciparum malaria

Although falciparum malaria shares with conditions such as severe bacterial disease much evidence for endothelial activation (eg adherence molecules [138,139] and circulating endothelial microparticles [140,141]) and its consequences (eg platelet and leukocyte adherence, above), it is distinguished from them by the presence of another obvious adherent object – erythrocytes containing mature parasites. From the biology of the erythrocytic phase of *P. falciparum*, vascular sequestration (endothelial adherence) of parasitized red cells, somewhere in the circulation, is inevitable for roughly the last half of the 48 hr erythrocytic cycle. Thus mature erythrocytic forms of the parasite are rarely seen in peripheral blood smears. This adherence was first noted in autopsy samples in the 19th century [142], and fuelled the widely (but not universally; see below) held view that much of the illness and pathology of this disease needed little explanation other than that offered through consequential impairment of microvascular flow. Thus, through the decades a common thread in proposals to explain falciparum malaria disease has been a primary role for tissue hypoxia caused by vaso-occlusion by parasitized red cells. The presence of coma, hyperlactataemia, hypoglycaemia, and metabolic acidosis, all three consistent with a patient being forced to rely on anaerobic glycolysis for energy production, have encouraged this viewpoint. As summarized earlier, cytokine-induced cytopathic hypoxia can also explain these phenomena.

#### Microparticles in systemic inflammatory diseases

Microparticles are an intriguing component of the inflammatory system. First described in 1967 [143], they are a heterogeneous group of small membrane-coated vesicles released from many types of cells upon their activation or apoptosis, but differ from apoptotic bodies. They retain at least some functions of their cell of origin, which can include platelets, endothelial cells, and various leukocytes. Triggers for their release include TNF [144], and they are increased in the circulation in systemic inflammatory states such as sepsis and trauma [145,146], as well as inducing inflammatory cytokine release themselves [147]. Along with cytokine increases, endothelial activation and the activation and adhesion of platelets and leukocytes, microparticles provide further evidence for a common pathophysiology of sepsis, trauma, and malaria [140,148,149]. While microparticles enhance the general inflammatory activity in these circumstances, and their exploration within the context of malaria is novel, evidence that they are likely to prove to be a key to controlling malarial disease, any more than other inflammatory conditions in which they act as markers of severity, is so far lacking.

### The interaction between cytokines and sequestration

#### Arguments against a primary role for sequestration in falciparum malaria illness

As noted earlier, sequestration is common in certain tissues of fatal cases of falciparum malaria. Nevertheless, a primary (ie prior to cytokine increase) harmful vaso-occlusive role for sequestered red cells containing parasites does not, in our view, withstand close scrutiny. As a simple practical example, it requires the pathophysiology in patients equally ill from uncomplicated falciparum malaria and vivax malaria to be quite different. Parallels between vivax malaria and the outcome of injecting TNF into human volunteers [75,150], and increased levels of this and functionally-related cytokines in vivax patient sera [151] plus the non-sequestering nature of the parasitized red cells, are consistent with cytokines being sufficient to cause the illness of vivax malaria. This includes the occasional, but well documented, coma (see below). Logically, therefore, these cytokines are sufficient to explain uncomplicated falciparum malaria, a condition notoriously difficult to separate clinically from vivax malaria [76]. This is consistent with occlusive parasitized red cells becoming an essential part of pathogenesis only when falciparum disease becomes more severe, and parasitized erythrocytes start to favour physiologically sensitive areas such as the cerebral vasculature. Alternatively, the total number of parasites, sequestered or otherwise, may, through the cytokine concentrations they generate, be what counts.

The erythrocytic life cycle dictates that sequestration is inevitably present before the onset of illness or in malaria tolerant individuals, yet no information seems available on it taking any particular anatomical pattern in these people. Newer technology involving expression of luciferase by a defined stage of the life cycle, to date applied only to a mouse model (where it revealed intriguing results that warrant reading closely [152]), would be impractical in man. However, an indirect appreciation of the avidity, if not the location, of *P. falciparum *sequestration in asymptomatic, malaria-tolerant individuals (usually not even fever, yet parasite densities overlapping those seen in severe illness) comes from work done in Mali [153], in which blood smears were made three time a day, usually at 6 hr intervals, for 12–13 days. Some 3000 blood smears were examined. Fluctuations in parasite density between consecutive smears often proved to be massive and abrupt, and the authors concluded that such change could come only from mirror-image changes in sequestration rate, not through parasite multiplication. Transitory disappearance of parasites from the peripheral blood occurred at least once in all infected individuals (63 of 79 subjects). Such slide negativity was of short duration, unrelated to the cycle of trophozoite maturation, and attributed to dramatic increases in sequestration of existing parasites. To our knowledge this rapid oscillation between circulation and sequestration is not reported during untreated malarial illness. On this evidence, avidity of sequestration is lower in malaria-tolerant individuals than in patients, suggesting different controlling influences, with sequestration in the former group, but not in the later, being independent of inflammatory cytokines.

At autopsy, brain is often a favoured site of sequestration, but whether this preference occurs before coma onset, or develops while coma is progressing, has not been determined. Marked brain sequestration at death has not been a universal observation, and, as summarized by Maegraith [18], reports have accumulated since the 1920s on a mismatch, at autopsy, between cerebral sequestration and coma. Such evidence is still being presented [71], as discussed below. Impaired consciousness can occur in a range of systemic inflammatory states, being present in certain viral and bacterial diseases as well as malaria. Thus regarding parasite sequestration as a necessary mechanism for falciparum malarial disease ignores its close clinical and pathological similarity, in terms of metabolic changes and organs affected, to other diseases that also can cause impaired consciousness, but lack parasitized red cells. These conditions are now accepted to be systemic inflammatory states [76]. As summarized below, the broader literature is consistent with our novel proposal (below) that, in addition to the above post-sequestration activity of inflammatory cytokines, these mediators will have earlier determined, during severe illness, where most sequestration occurs.

For some time most adherents of the traditional sequestration-based view of falciparum malaria disease have accepted what they regard as secondary roles for inflammatory cytokines in falciparum malaria disease [154]. By these tenets, sequestration at sensitive sites, such as brain capillaries, leads to higher local concentrations of these cytokines near sequestered parasitized red cells, since these are the source of the parasite material that triggers their release, and endothelial cells and leukocytes are commonly their source. The importance of the site of sequestration to disease severity is thereby amplified, involving both vaso-occlusion and secondary cytokine effects, such as increased blood-brain barrier permeability [155]. This as a plausible aspect of the relationship between sequestration and inflammatory cytokines in falciparum malaria, but the concept outlined below is proposed here to be their major interaction in falciparum malaria.

#### Influence of inflammatory cytokines on organ distribution of sequestration

By far the bulk of the literature arguing for occlusion-induced pathology in falciparum malaria concerns its documentation in the brain [156] and placenta [157] in sick individuals, and it is timely to consider why these sites are favoured. Equally, why are these the main sites where monocyte accumulations occur? The following explanation seems highly plausible, and is testable. In brief, it suggests that sequestration favours the brain when circulating concentrations of TNF are high (ie when the patient is ill), but not before onset of illness, or in malaria tolerant individuals. The absence of mature forms on blood smears from these two groups implies that the capacity of parasitized red cells to sequester is quite robust, but sequestration is not focussed at harmful sites without raised inflammatory cytokines.

For over a decade there has been substantial evidence that inflammatory cytokines (TNF the most studied) increase expression on endothelial cells of the molecules to which parasitized erythrocytes adhere [158-160]. Being driven by cytokines whose detection and concentrations correlate with degree of illness, this increase can be expected to operate only in moderate to severe illness, not early in infection, when TNF levels are undetectable, or in tolerant individuals, who have been argued to be refractory to malaria-induced TNF [161]. Blood smears confirm that sequestration occurs in these individuals. In the next paragraphs it is argued that during severe falciparum illness these cytokines (provided they have not killed the patient beforehand) could concentrate most sequestration to the sites familiar at autopsy.

TNF and interleukin-1 increase tissue factor expression on endothelial cells and mononuclear cells [162], thereby initiating pathways that generate thrombin (reviewed recently [163]), a molecule with many roles at the cross roads of inflammation and coagulation. When bound to thrombomodulin, a thrombin receptor on the endothelial cell surface, thrombin activates protein C, which can degrade Factor VIIIa and Factor Va, essential cofactors in the activation of Factor X and prothrombin respectively [164]. These feedbacks play a central role in keeping coagulation in homeostasis.

**Figure 1:**
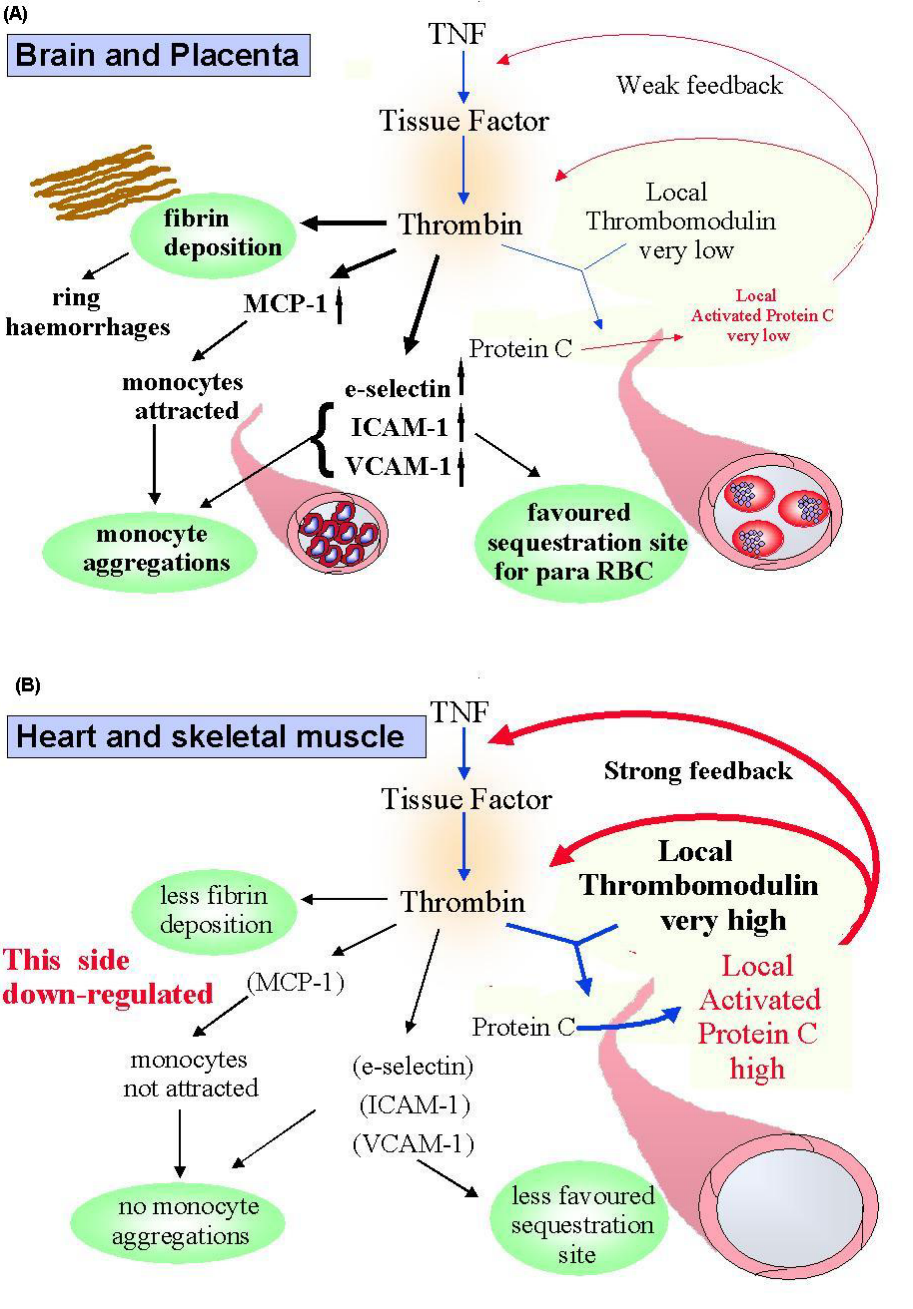
The proposed influence of differences in thrombomodulin levels on cytokine-induced expression of adhesion molecules on endothelial cells, and monocyte attraction, in different organs. (a) tissues with low endothelial thrombomodulin levels (b) tissues with high levels.

It follows, therefore, that tissues in which thrombomodulin density on endothelial cell surfaces is lowest (brain least – indeed reported undetectable in an earlier study [165] – placenta next least, and other organs more [166]), will have more unbound thrombin left available for its other functions on activated endothelium. These include a ten-fold upregulation of adhesion molecules such as E- selectin by TNF [167], CD36 [122], intercellular adhesion molecule-1 (ICAM-1 CD54) and vascular cell adhesion molecule-1 (VCAM-1 CD106) [168] and, with implications for the observed accumulation of monocytes, monocyte chemotactic protein-1 (MCP-1) [169]. Moreover, thrombin – thrombomodulin complex formation will be low in tissues where endothelial thrombomodulin is low. Therefore protein C activation will be correspondingly low, and the negative feedback that controls TNF-induced thrombin formation correspondingly weak (Figure 1a), further enhancing concentrations of the above adhesion molecules. Levels of a range of inflammatory cytokines, including TNF, are high in supernatants of villous leukocytes from malarial placentas [170], so these principles should apply to the monocyte accumulations and heavy sequestration in this organ also.

**Figure 2:**
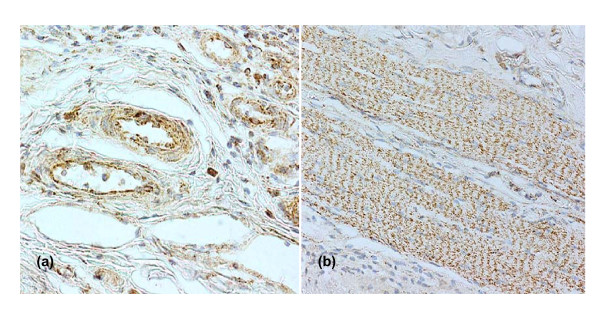
Immunohistochemical staining of the gut wall of malaria patients to detect iNOS. Techniques (DAB, haematoxylin), materials and controls as in reference 71. Cases (a) MP6 and (b) MP21 (see Table 1 of ref. 71) are shown. Unpublished data.

Thrombomodulin also sequesters HMGB1, making it less available to activate RAGE (the receptor for advanced glycation endproducts, shared by this cytokine [171]), so it cannot express its full inflammatory potential [172], and thus generate a further wave of cytokines such as TNF. Hence a given concentration of HMGB1, a cytokine increased in serum in sepsis [53] and falciparum malaria [57] in proportion to degree of illness, can be predicted to exert more pro-inflammatory influence in brain and placental vessels, where more of it is functionally available because less of it sequesters on thrombomodulin. Unfortunately the intestinal blood vessels, another favoured site for sequestration in falciparum malaria, were not included in either the CD36 [122] or the thrombomodulin study [166]. The reverse of the arguments for brain serve to rationalise why sequestration is rare or absent in heart and skeletal muscle, tissues at the other (high) end of the thrombomodulin spectrum [166], and therefore with least free thrombin left available to upregulate sequestration sites during TNF-induced illness (Figure 1b). For these reasons harmful sequestration is best regarded as a consequence of increased inflammatory cytokine generation, as well as a potential way to focus release of these mediators at sequestration sites. In short, differential endothelial activation induced by high levels of circulating inflammatory cytokines could shift the emphasis of sequestration to potentially harmful locations where, through schizogony, it could then initiate the bulk of the next wave of cytokine release. As previously noted for neuron function in malaria [173], nitric oxide generated by iNOS induced by these cytokines could also plausibly explain the small intestine intussusception seen in most children dying with malarial coma in Malawi [174]. Nitric oxide has an essential role in an experimental model of this pathology [175], and iNOS is strongly induced in the small vessels of the jejunum (Figure 2) in this patient series.

### Anaemia

As recently reviewed [176], critical illness associated with an inflammatory response invariably causes multifactorial anaemia. It has often been noted that anaemia could contribute to poor oxygenation of tissues in malaria [177] and there is general acceptance that it can be severe enough to reduce supply of oxygen to mitochondria to dangerously low levels. Thus it can be a major component of malarial pathology. Obviously a high parasite load indicates imminent widespread lysis, but anaemia does not correlate with parasitaemia, and sometimes is extreme when very few parasites are present.

#### Poor red cell deformability

Erythrocytes have a limited life, determined by how long they can remain flexible enough to squeeze through fenestrations in specialised vessels in the red pulp of the spleen [178]. A red cell that cannot pass this test is phagocytosed by adjacent macrophages, and lost. In health this loss is balanced by erythropoiesis, and haematocrit remains normal. Should red cells develop a premature poor deformability they are removed from the circulation correspondingly earlier.

Like other cells, erythrocytes stay intact by constantly extruding Na^+^ in exchange for K^+ ^through an energy-dependant "pump" in their cell membrane that was defined by the ability of certain digitalis gylcosides to block it. This Na^+^/K^+ ^pump fails, and intracellular Na^+^ accumulates in (non-parasitized as well as parasitized) red cells during human [179] or monkey [180] malaria. These changes in ionic content of red cells have been observed in a sepsis model [181]. In another sepsis model [182], erythrocyte deformability could be shown to be caused by NO, an inhibitor of this membrane pump [183]. Since inhibition of the Na^+^/K^+ ^pump *in vitro *correlates with a reduced red cell deformability plus a parallel decrease in red cell filterability [184], any influence, such as NO [183,185], that inhibits this pump could potentially cause poor red cell deformability. Cytokine-induced iNOS provides a demonstrable [71] way for these changes to occur in severe malaria.

Originally recorded in uraemic patients, poor red cell deformability was observed in a small pilot study of malaria patients in 1985 [186]. Soon after it was recognized in sepsis [187,188], and subsequently studied in falciparum malaria with a view to understanding both circulatory obstruction [189] and anaemia [190]. There is good evidence that, when measured on admission, a severe reduction in red cell deformability is a strong predictor of malarial mortality [189], but whether this is cause and effect, or the two phenomena are simply inevitable co-travellers in a strong pro-inflammatory milieu, is unclear. It seems clear that poor red cell deformability (which affects parasitized and unparasitized red cells equally) and dyserythropoiesis can lead to severe anaemia in various diseases, particularly in chronic infections such as malaria. Its presence in vivax malaria [191] implies that its role in vaso-occlusion is less important.

Clearly, it would be useful if a shortened lifespan of red cells through their premature loss of membrane flexibility were compensated for by a faster rate of erythropoiesis. Unfortunately, the inflammatory cytokines that shorten the lifespan of the red cells also slow down their replacement, as outlined in the next section. Their combined effect on reducing haematocrit can be expected to be rapid.

#### Dyserythropoiesis

Because the parasite inhabits erythrocytes, which must burst if the parasite is to propagate, the obvious initial conclusion was that this source of red cell loss was central to the fall in haematocrit seen in this disease. As reviewed nearly 60 years ago [192], this fall was soon realised to be out of proportion to the number of red cells parasitized, so other factors were realised to contribute. Phagocytosis of unparasitized red cells was also recorded decades ago in monkey [193] and human [192] malaria, and for many years was regarded as sufficient explanation for this discrepancy. Others had been investigating dyserythropoiesis in the bone marrow of patients with falciparum malaria [194,195] and stressed its contribution to malarial anaemia. A group in Oxford [196], seeking an explanation for this dyserythropoiesis through an electron microscopy study of bone marrow, observed sequestration of parasitized red cells and argued that this caused the bone marrow dysfunction in falciparum malaria by restricting blood flow and thus inducing hypoxic changes. This idea proved inadequate, however, when this same group subsequently reported dyserythropoiesis and erythrophagocytosis in vivax malaria, in which parasitized red cells do not sequester [197].

Twenty-five years ago our group proposed that TNF might cause the bone marrow depression seen in malaria [15]. Subsequently an undefined product in macrophage supernatants [198], later identified as TNF [199], was found to inhibit the growth and differentiation of erythroid progenitor cells. When rTNF became available (but before it had become technically possible to assay for this cytokine in human serum) the dyserythropoiesis and erythrophagocytosis seen in terminal *Plasmodium vinckei*-infected mice were reproduced when a single injection of rTNF was given early in the course of the infection [200]. Phagocytosis of erythroblasts in bone marrow, a phenomenon also reported by Wickramasinghe *et al*. [196,197] in human malaria, was commonly observed [200]. Decreased erythropoiesis was subsequently reported in mice receiving continuous TNF infusions via implanted osmotic pumps, and increased erythropoiesis in malarial mice after injecting neutralizing antibody directed against murine TNF [201]. TNF-induced dyserythropoiesis has since been confirmed in rats [202], and mice expressing high levels of human TNF become markedly anaemic during malaria infections [203], even though parasite numbers, and therefore red cell loss post-schizogony, are considerably reduced.

The past decade has seen an expansion of this line of enquiry into human malaria, and also the number of cytokines, both pro-inflammatory and anti-inflammatory [204,205] in absolute amounts and ratios [206,207], that have been investigated in this context. It has been extended to include other pro-inflammatory cytokines, such as IL-12 [208] and FasL [40], and the role of the persistence of production of such cytokines in the anaemia of falciparum malaria infection has recently been examined [209]. Suppression of prostaglandin E2 during malaria infection has also been shown to have an important influence on these events [210].

A decade ago the mechanism of TNF-induced damage to human bone marrow cells was argued to be nitric oxide generated by iNOS induced by TNF [211]. More recently attention has focussed on another cytokine, MIF, downstream of TNF but also induced by agents other than TNF, as a cause of malarial dyserythropoiesis. Martiney and co-workers [212] found that MIF was enhanced in a mouse malaria model, and that rMIF inhibited the formation of erythroid (BFU-E), multipotential (CFU-GEMM), and granulocyte-macrophage (CFU-GM) progenitor-derived colonies *in vitro*. Subsequently, MIF proved to be strongly detectable by immunohistochemistry in systemic, but not cerebral, vascular smooth muscle of fatal African paediatric sepsis and falciparum malaria [71]. It has very recently been found that sub-inhibitory concentrations of MIF synergise profoundly with TNF and interferon-gamma in inhibiting mouse erythroid precursor colonies [213]. These authors provide other data that greatly strengthens the case for a major role for MIF in malarial dyserythropioesis. This work provides a timely warning against the reductionist approach to understanding the actions of cytokines in disease, which does not reflect the *in vivo *reality of a considerable number of these mediators being present simultaneously. Thus the slow replacement rate of red cells in malaria through the influence of inflammatory cytokines is now a well-established aspect of malarial disease pathogenesis. In summary, cytokines induced by malaria products are a major determinant of haemoglobin deficiency, and thus the rate at which oxygen reaches mitochondria in malaria.

Infection-induced dyserythropoiesis is not restricted to malaria. The first awareness of it in other infectious diseases appears to have been its description in HIV patients, plausibly as a consequence of opportunistic infections [214]. It has subsequently been observed in acute viral hepatitis B [215], simian [216] and human [217] parvovirus B19, visceral leishmaniasis [218] and dengue [219], all conditions that are associated with increased levels of circulating TNF, and doubtlessly its regulators, such as MIF. As noted above, the effect on red cells of the combination of a lower rate of production and accelerated destruction can be expected to lead to severe anaemia. The literature on both these influences on red cells underline how widely the consequences of excessive inflammatory cytokines impinge on disease pathogenesis, and emphasise the conceptual limitations imposed by regarding falciparum malaria as somehow outside the sphere of these host-origin mediators [5,25].

### Cardiac insufficiency

Cytokine-induced myocardial depression frequently accompanies severe sepsis (see [220]). Whereas it was previously considered a pre-terminal event, it is now clear that cardiac dysfunction, as evidenced by biventricular dilatation and reduced ejection fraction, is present in most patients with severe sepsis. It has been known for some time to be caused by soluble factor(s) released by macrophages exposed to endotoxin [221]. Once cloning of cytokines occurred its activity was attributed to IL-1, then also to TNF [222], then the two synergistically [223], and finally to IL-6 [224], a macrophage product induced by both of these mediators. A literature exists on these effects being minimised by blocking MIF, which reduces the feedback inhibition of TNF production by glucocorticoids [225,226]. As discussed in above, excess inflammatory cytokines have been shown to cause cardiomyocyte mitochondrial dysfunction. Since TNF [38], IL-1 [38], IL-6 [39] and MIF [71] are all highly expressed in falciparum malaria, it can be expected that these cardiac-depressing activities would be acting in this disease as well as in sepsis. Evidence of this, in terms of circulating cardiac proteins, has accumulated in the past few years [227,228], although its clinical impact is yet to be evaluated. It may be present, but its potential clinical impact is over-ridden by the effects of hypovolaemic shock, as summarized in the next section.

### Poor circulating volume

Insufficient intravascular volume is ultimately of concern in disease because, through poor perfusion, it leads to poor oxygen supply where it matters, the microcirculation that feeds the mitochondria within the cells that form the tissues the capillaries pass through. The major therapeutic option is volume resuscitation. As recently reviewed [229], it is under the control of a number of autoregulatory mechanisms, and these are known to be disrupted in sepsis. As well as the effects of changed red cell deformability, and adherence of platelets and leukocytes, as discussed above, variation in iNOS induction, leading to more or less nitric oxide, essential for local degrees of the vasodilation that perfusion depends on, are major controlling factors.

Using a range of indicators, workers in Kenya have confirmed the older observation [230] that shock is not rare in severe falciparum malaria [231], and that the haemodynamic changes in children with severe malarial anaemia complicated by the respiratory distress were more characteristic of hypovolaemia than of biventricular failure [232]. They have also demonstrated that while administering albumin did not improve acidosis, it did reduce mortality [233]. This conceptual approach has been strenuously questioned by others who detected only a mild fall in total body water volume and extracellular water volume [234], as well as the relative rarity of severe hypotension in falciparum malaria compared to the shock that can accompany trauma or sepsis [4]. However, local effects may be much more important in falciparum malaria than in sepsis – for example these could in part arise from vasodilation being much more uneven in malaria than sepsis because of patchy local foci of post-schizogony malaria toxin release from sequestered parasites, and local generation thus of the inflammatory cytokines that induce endothelial iNOS [71]. It is recognized that during treatment one would need to be cautious of fluid overload if the patient displays evidence of cerebral oedema [235] or cardiac insufficiency [236].

The detailed arguments on both sides of this debate are beyond the scope of this review, except to note that they are currently a major fault line between those who propose that malaria has a fundamentally similar pathophysiology to other acute systemic infections [21,233,237] and those who see it as unique [238]. This issue cannot be resolved until recognition is given to the need to research the pathophysiology of malaria and other systemic infectious states in parallel rather than, as at present, in isolation.

**Figure 3:**
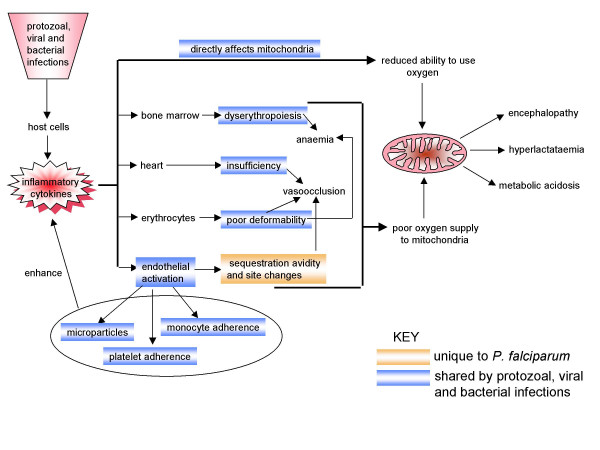
Proposed control of pro-inflammatory cytokines over the influences that limit oxygen delivery, as well as over the capacity of mitochondria to use oxygen.

From this and the previous section, it is not hard to visualise the combined harmful effect on the patient when systemic inflammation reduces oxygen supply to their cells also makes these cells worse at using it. As shown in Figure 3, the initiating pathophysiological lesion is the onset of the systemic inflammatory response, and it is difficult, from the evidence, to envisage sequestering parasitized red cells, *per se*, initiating malarial disease before it is focussed to sensitive organs by systemic release of inflammatory cytokines. Sequestering parasitized red cells may then, in part through locally released cytokines, exacerbate the illness if the patient survives long enough.

## Practical consequences of these changes

### Hyperlactataemia in malaria and other infectious diseases

Hyperlactataemia, a recognized marker of falciparum malaria severity, is at the centre of controversies relevant to the theme of this review. Its discussion requires some basic biochemical background. The lactate anion has complex roles in biology. Hyperlactataemia may be associated with acidosis, a normal pH, or alkalosis [239], and can occur in viral and rickettsial diseases [240], as well as (see below) sepsis and malaria. In synopsis, most lactate is generated during glycolysis, which essentially consists of oxidising glucose, a six-carbon structure, into two three-carbon molecules of pyruvate. This is reduced to lactate through the action of pyruvate dehydrogenase, a reaction that avoids pyruvate accumulating, and supplies NAD^+ ^to keep glycolysis going. Thus lactate can be formed as a byproduct of glycolysis, which can occur in all metabolically active tissues and supplies ATP, albeit in small amounts, independently of the presence of oxygen. Every mole of glucose metabolised by anaerobic glycolysis to carbon dioxide and water yields 4 moles of ATP, whereas oxidative phosphorylation within mitochondria yields 32 moles of ATP. When oxygen usage falls (whether through poor supply or poor utilisation) ATP generation falls, and glycolysis is accelerated to compensate, as much as possible, for this energy loss. A consequence is oversupply of the byproduct, lactate, but from a disease perspective this is a side issue compared to insufficient ATP generation, even though the two may correlate well. Enhanced glycolysis under aerobic conditions can also increase lactate production. The metabolic acidosis secondary to this failure of mitochondrial energy production, which high lactate often accompanies, is a consequence of this energy failure, and inevitably accompanies it in severe inflammatory illnesses, including malaria and sepsis.

The body's supplies of glucose, including stores of its polymer, glycogen, are not unlimited, so when glycolosis is enhanced for any period it sooner or later runs out of fuel. Gluconeogenesis fills the breech as much as possible, but it soon fails because substrate supplies are limiting [241]. These events are reflected in the hypoglycaemia that has often been reported in severe malaria [242] and sepsis [243,244]. When seen in this context hypoglycaemia in these diseases is no longer a primary cause of harm, such as when driven by hyperinsulinaemia, but an inevitable consequence of over-exuberant, typically anaerobic, glycolysis.

#### Origin of the high circulating levels of lactate

##### Poor oxygen delivery to mitochondria

In the 19th century an association had been noted between hypoxia and lactate accumulation in tissues, and a progression of logic through the physical exercise literature [245] led to lactate levels being seen not only as a marker for poor oxygen delivery in disease states, but also a consequence of it, and the cause of the acidosis. For some time hyperlactataemia has been regarded as a functionally relevant marker for a poor prognosis in both sepsis [246] and malaria [247]. It is now well accepted that hyperlactataemia correlates with a poor prognosis in paediatric falciparum malaria in Africa [97,177,248]. Although the sepsis world now discusses several origins for the lactate increase, including inflammation-induced mitochondrial dysfunction [103], in falciparum malaria it is still generally attributed to a reduced oxygen supply, mostly through microvascular occlusion by sequestered parasitized erythrocytes [70]. Unfortunately, the traditional conceptual approach (in which not only are acid properties attributed to the lactate anion but lactate and lactic acid are equated and used interchangeably) is dying hard in the malaria world.

Recent publications [4,238] promote the traditional view by arguing that lactate/pyruvate ratios are higher in malaria than in sepsis (but see [249]), and can therefore be explained only by hypoxia through vaso-occlusion [4,70]. However, it seems difficult to envisage a mechanism whereby insufficient oxygen reaching tissue mitochondria would generate higher lactate/pyruvate ratios than would its poor utilisation once there. Indeed an inadvertent positive control of mitochondrial dysfunction, seen as the side effects of treatment of HIV patients with nucleoside reverse transcriptase inhibitors that cause mitochondrial toxicity [250], can generate lactate/pyruvate ratios up to double that recently reported for severe malaria [4,70], and as high as any value reported for the severest adult cases in Thailand [251].

In order to test the possibility that sequestration is essential for these changes in skeletal muscle sections, tissues previously stained for other purposes [71,252] have been recently re-examined for adhering parasitized red cells. Since skeletal muscle is a tissue with one of the highest rates of oxygen consumption [253], it is predictably a large generator of lactate when anaerobic respiration dominates, whether triggered by oxygen insufficiency from vascular occlusion by sequestering parasitized erythrocytes or its adequate presence, but under-utilisation, through mitochondrial dysfunction. To address the vaso-occlusion possibility, 1–3 tissue sections from chest wall and/or diaphragm from each of 27 previously described [71] fatal malaria cases of African paediatric malaria were examined blind by three observers. Negligible sequestered parasites were observed in 24 of 27 cases (unpublished data), including 15 displaying a 3+ or more sequestration score in cerebral capillaries. In the light of the high thrombomodulin levels on endothelial cells in vessels in skeletal muscle [166], and its implications for expression of adhesion molecules (Figure 1b), this is not surprising. Unfortunately, lactate assays were not performed on this patient series. Nevertheless the high incidence of hyperlactataemia in fatal cases from this same population [254] implies that the incidence of skeletal muscle sequestration would need to be considerably higher than observed for this hyperlactataemia to have its origins in impeded oxygen delivery to this main glucose-consuming organ.

##### Poor oxygen usage by mitochondria

The concept termed cytopathic hypoxia [103], is now considered to be a major contributor to the pathogenesis of sepsis. This is consistent with the evidence from animal models that neutralizing TNF prevents [255] or reverses [256] metabolic acidosis in experimental sepsis, although the indirect action of TNF on oxygen delivery, through its effects on endothelial activation, and thus platelet and leukocyte adhesion, can also be expected to contribute *in vivo*. In addition, inhibiting TNF successfully treated the metabolic acidosis of sepsis in a double blind trial in premature infants [257], and immunizing mice against GPI, a malarial toxin selected for its capacity to induce TNF production, inhibited metabolic acidosis in a mouse malaria model [65]. These outcomes are equally likely to apply in any disease in which levels of the pro-inflammatory cytokines, including TNF, are raised, and metabolic acidosis occurs. Studies on muscle ATP depletion during severe sepsis in patient material [258] and experimental animals [259] have provided data consistent with these arguments. It would be most instructive to repeat these same experiments with muscle biopsies from malaria cases.

Since the inflammatory cytokines that cause mitochondrial shutdown are prominent in both sepsis and malaria [38,39] it can be inferred that this organelle dysfunction is an equally plausible cause of reduced ATP synthesis and increased lactate accumulation in both diseases, correspondingly diminishing the need to invoke the argument based on parasite-induced vascular occlusion to explain these changes in malaria. Moreover, the mitochondrial ultrastructural damage that correlates with lowered oxidative phosphorylation in a sepsis model [260] parallels that described by Maegraith in monkey malaria [261]. In recent decades few malaria researchers have remarked on the demonstration, by Maegraith's group, decades ago, of the now topical inhibition of mitochondrial function in malaria [262,263]. In our opinion these were landmark observations that paved the way for a role for severe systemic inflammation in causing the metabolic acidosis and contributing to the raised lactate levels seen in severe malarial disease. Indeed, in 1954 this group discussed the concept of functional hypoxia in malaria arising from interference with oxygen acceptance by tissue cells [264].

#### Hypoxia and TNF both induce HIF-1

Hypoxia-inducing factor-1 (HIF-1) is a transcription factor that regulates activation of several genes responsive to low oxygen, including erythropoietin, vascular endothelial growth factor, glycolytic enzymes, and glucose transporters. These pathways need to be switched on whenever aerobic respiration is reduced, since they are essential for the body to generate as much ATP as it can. As evidence that both poor oxygen delivery and its poor utilization through the inhibitory effect of inflammatory mediators on mitochondria produce the same functional and, therefore, clinical end results, it should be noted that TNF [265] has, even in normoxic cells, the same HIF-1-inducing ability as has hypoxia [266]. This is predictable from the ability of TNF to shut down mitochondria (eg via the reversible effect of nitric oxide on cytochrome c [104]), the oxygen sensor that regulates HIF [267]. It provides a plausible explanation for the accelerated rate of aerobic glycolysis sometimes reported in sepsis [268], which can increase pyruvate and lactate levels in the absence of hypoxia [269].

Should cytokine-accelerated glycolysis occur under aerobic conditions, any resultant hyperlactataemia cannot be expected to be associated with acidosis, since protons are generated by ATP hydrolysis in mitochondria, and pH remains constant. The desirability of enhancing HIF-1 in systemic inflammatory states, in which category the presence of excess pro-inflammatory cytokine production places malaria, has recently been reviewed [270]. MIF, a pro-inflammatory mediator shown to be upregulated dramatically in a number of tissues in both severe falciparum malaria and sepsis [271], also accelerates glycolysis [272], so can be expected to contribute to the hypoglycaemia and hyperlactataemia of both diseases. As with HIF-1, both hypoxia [273] and TNF [42] upregulate MIF. Since anti-MIF antibody prevents hypoglycaemia and increases fructose 2,6-biphosphate in TNF (-/-) mice administered endotoxin, MIF is argued to act independently of TNF [42]. The relative importance and interaction of HIF-1 and MIF in this context have not yet been examined, but these activities of TNF and MIF nevertheless stress that an understanding of malarial disease needs a broader vision than simple vascular occlusion.

#### Metabolic acidosis

Metabolic acidosis is not a disease, but a symptom of a serious underlying process. As recently summarized [274], metabolic acidosis is defined as a decrease in blood pH secondary to a decrease in the bicarbonate concentration. The decrease in bicarbonate concentration may be secondary to an excess of acids that will consume bicarbonates, reflecting the open character of this buffering system, or to a loss of bicarbonates through the digestive or the renal route. These authors [274] note that importance of the metabolic acidosis may be appreciated by measuring the change in bicarbonate concentration from the normal, or the base excess, which gives a better assessment of the acid load because it takes into account the buffering of the non-bicarbonate systems. Despite the impression that much malaria literature gives, metabolic acidosis is not unique to this disease, being seen in viral, rickettsial and bacterial infections [240] as well as acute gastroenteritis, where its prevalence is higher than in malaria [275].

##### Lactate: a cause, marker, or neither in the acidosis of malaria or sepsis?

The notion persists in some malaria circles that excess lactate accumulation causes the metabolic acidosis that correlates with a poor clinical outcome, and therefore warrants therapeutic reduction [4,276]. Accordingly, these authors argue that lowering lactate levels with sodium dichloroacetate (DCA), an inhibitor of pyruvate dehydrogenate kinase, would be sufficient to ameliorate the metabolic acidosis of falciparum malaria [4,276,277]. In our view the current literature on systemic inflammatory diseases does not support this. To successfully treat metabolic acidosis DCA would need to increase oxygen consumption so that oxidative phosphorylation takes over from anaerobic glycolysis as the dominant ATP producer, and the protons generated when ATP is resynthesized from ADP and inorganic phosphate are re-consumed, increasing pH to normal levels [278].

A recent editorial in Critical Care Medicine [279] has lucidly summarized the key points of the mechanism of metabolic acidosis in sepsis, a condition that shares systemic inflammation and a range of its consequences with severe malaria (Table 2). These authors do not accept that lactate is the cause of the acidosis associated with hypoxia. Instead, they note the evidence that cellular acidosis during hypoxia, be it from limited oxygen supply or utilisation, arises from the hydrolysis of non-mitochondrial ATP. Every time a molecule of ATP undergoes hydrolysis, a proton is released. When oxygen is readily available, the products of this reaction, including protons, are recycled by mitochondria, and pH does not change. During hypoxia, however, the mitochondrial turnover rate drops below the rate of ATP hydrolysis, so protons are being produced faster than they can be recycled, and intracellular pH falls once buffering capacity is exceeded. Since these reactions are independent of lactate levels, merely reducing the level of this anion can therefore no more be expected to increase survival rate in falciparum malaria than it did in sepsis [280]. Indeed, it could in theory harm comatose patients, since there is evidence that lactate helps brain tissue survive hypoxic and hypoglycaemic episodes [281-283], and the lactate shuttle is proving to be how astrocytes protect neurons from metabolic stress [284]. Moreover, infusing enough lactate into patients with severe sepsis to cause hyperlactataemia did not cause acidosis, but an alkalosis [285].

**Table 2:** 

	**Influenza encephalopathy**	**Cerebral malaria**
seizures/coma after high grade fever	+	+
metabolic acidosis	+	+
hyperlactataemia	+	+
serum TNF, IL-6, sTNFRI up	+	+
serum nitrite/nitrate up	+	+
CSF TNF, IL-6, sTNFRI up	+	+
multiple organ failure, sequelae	+	+
thrombocytopaenia	+	+
damage to vascular endothelial cells	+	+
brain oedema/damage to BBB	+	+
apoptosis in neurons/glial cells	+	+
evidence of active caspase-3 (brain)	+	+
caspase-cleaved PARP (brain)	+	+

Even when considerable lactate is generated under these conditions, it is now evident that other anions contribute much more than it does to the strong ion difference (SID) that, through influencing the body's buffering capacity, has a considerable influence on acidosis in sepsis [286,287] and falciparum malaria [275,288]. Thus lactate, while to some degree a useful marker of metabolic acidosis in malaria and sepsis, seems not to be a cause of it, and therefore not a worthwhile therapeutic target.

The basic biochemistry of these pathways, in which protons are consumed when ATP regeneration occurs aerobically, but accumulate, lowering the pH, when ATP regenerates anaerobically, was elegantly set out by Dennis in 1991 [289]. Others have noted the difficulty in separating the effects of acidosis from those of hypoxia. Since much evidence points to hypoxic cells preferring an acidotic environment [274], they question the wisdom of unthinkingly reversing acidosis. Establishing whether increased circulating lactate levels merely indicate a dominance of anaerobic glycolysis over oxidative phosphorylation in cellular energy generation, or whether it is innately harmful because its formation generates acidosis during oxygen-independent glycolysis, clearly has treatment implications.

##### Effect of cytokine-induced defective respiratory muscle function on acidosis

The importance of hyperventilation in helping to get rid of the excess CO_2 _that accumulates in metabolic acidosis, inactivating the bicarbonate buffering system, is well accepted in sepsis [290], and mechanical ventilation is used, as necessary, to facilitate its removal. The decrease in blood pH will depend on the PaCO_2_, which reflects the respiratory response to the metabolic disorder. As reviewed [274], if the metabolic acidosis is isolated and the patient can breathe unassisted, the importance of the hyperventilation is predictable, and simple formulas (the most common used being: PaCO_2 _= 1.5 [HCO_3-_] + 8) give the PaCO_2 _from the HCO_3 _value. This has a parallel in the clear lung severe respiratory distress of falciparum malaria, with deep stressful breathing, first described in paediatric patients in coastal Kenya a decade ago. This hyperventilation predicted over three times the likelihood of a fatal outcome than did coma alone, and was recognized as a consequence of the underlying severe metabolic acidosis [236,291]. Logically this should prove to be associated with high circulating levels of pro-inflammatory cytokines.

**Figure 4:**
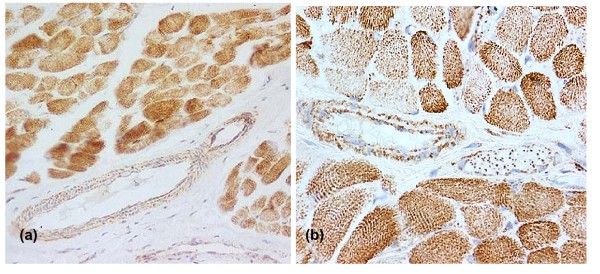
Immunohistochemical staining of (a) chest wall and (b) diaphragm muscle of malaria patients to detect iNOS. Techniques (DAB, haematoxylin), materials and controls as in reference 71. Cases (a) MP21 and (b) MP39 (see Table 1 of ref. 71) are shown. Unpublished data.

This need to breath heavily for an extended period demands much work of the muscles of the chest wall and diaphragm. Unfortunately, the pro-inflammatory cytokines that contribute to the acidosis also generate the inducible form of nitric oxide synthase (iNOS) in skeletal muscle in animal models of sepsis [292] and patients with this condition [117]. This is associated with poor muscle contractility, and thus a reduced capacity to self-correct acidosis through deep breathing. There is evidence to link this causally with inflammatory cytokine-induced nitric oxide and oxygen-derived free radicals causing mitochondrial dysfunction within the muscle of the diaphragm. This is in keeping with a similar functional role for the strong iNOS staining (Figure 4a) in chest wall skeletal muscle of fatal malaria and sepsis cases in African children [71]. Diaphragm skeletal muscle (unpublished) show the same picture (Figure 4b). Intense staining of skeletal muscle for iNOS in sepsis [293] and malaria [71] is consistent with the large difference recently found in nitrite/nitrate levels in the same tissue between survivors and non-survivors admitted into intensive care because of sepsis [258]. These samples were subsequently analysed paramagnetically, and changes consistent with a decreased concentration of mitochondrial Complex I iron-sulphur redox centres, which are particularly susceptible to nitric oxide, were linked to a fatal outcome [294].

More recently, the function of mitochondria in the diaphragm muscle of rats treated with bacterial endotoxin, which induces, in man as well as rat, the range of pro-inflammatory cytokines raised in both sepsis and malaria, has been examined in detail. A range of genes for proteins required for the mitochondrial electron transport chain are downregulated [295], resulting in reductions in various key subunits of Complexes I, III, IV and V of the transport chain, and a large fall in mitochondrial oxygen consumption [296]. Energy production and thus work ability of skeletal muscle is decreased. New data from these workers' laboratory provides a rationale, in terms of preventing this mitochondrial dysfunction, for the haemoxygenase-1 (HO-1) has been reported to be greatly increased in key tissues in falciparum malaria and sepsis [252]. In brief, they found that administering an HO-1-inducing agent, haemin, before endotoxin, protects mice against endotoxin-induced mitochondrial dysfunction in diaphragm and cardiac muscle, including failed ATP generation [297]. They note the possibility of adopting this principle, if not this agent, in treating severe sepsis. Malaria is an obvious extrapolation worth exploring.

In summary, lactate is an approximate, but workable, marker for metabolic acidosis, which in turn is a good marker for hypoxia, the primary pathophysiological lesion in these diseases. Whether arising from (a) poor supply of oxygen to mitochondria (through vasco-occlusion, low circulating volume, anaemia or cardiac insufficiency) or (b) reduced cellular utilisation of oxygen through mitochondrial dysfunction (in response to severe systemic inflammation) the outcome is essentially the same. The presence of inflammatory markers and the nature of the illness implies that mitochondrial dysfunction is the constant, and poor oxygen delivery the variable, in a synergistic whole.

### Neurological involvement in malaria

#### Severe falciparum malaria – more than cerebral malaria

The literature on this disease reflects the fact that by far the most cases occur in children in tropical Africa, although comparisons with the clinical picture in Papua New Guinea, where children are said to experience a generally milder disease, [298] have been made. Over 60 years ago it was noted that the disease closely resembles that caused by fulminant bacterial or viral infections [299]. The clinical picture typically consists of three overlapping syndromes (impaired consciousness, severe respiratory distress, and severe anaemia), and the more of these present the higher the mortality. This complexity has been most clearly set out in the well-known Venn diagram based on observations made in coastal Kenya [300]. By using the term malaria with impaired consciousness, rather than cerebral malaria, the review from which this diagram is derived [300] avoids the pitfall of assuming that the altered brain function that can occur in severe falciparum malaria requires local histologically detectable changes that are unique to this condition. The other two syndromes common in this disease in African children, severe respiratory distress, an indicator of an underlying metabolic acidosis, and severe anaemia, are discussed below.

#### Cerebral vaso-occlusion and impaired consciousness in falciparum malaria

Cerebral malaria is certainly simpler to understand if one operates from the premise that, as Marsh and Snow so eloquently put it, severe malaria equals cerebral malaria equals cerebral microvascular sequestration [300]. Unfortunately, scientific argument on this question is muted, since publications are more readily obtained by dissecting the mechanism of sequestration than by questioning its importance. It is proposed here that, rather than consider the effects of systemic inflammation and cerebral vascular obstruction by sequestered parasitized erythrocytes as opposing theories of cerebral malaria (as the literature has until now), they are functionally inseparable, with harmfully located sequestration viewed as one of the many consequences of systemic inflammation. In addition, there are ample arguments against cerebral sequestration acting in isolation as a major cause of the coma of falciparum malaria.

Certainly coma can be a striking component of severe falciparum malaria, and sequestration plus accompanying microhaemorrhages a spectacular aspect of terminal brain histology. Yet, one without the other has commonly been reported, and a causal link questioned. An example from 60 years ago is by Kean and Smith [301], who collected 12 cases with clinical courses of cerebral malaria, yet cerebral vessels recorded as not being plugged. Another five cases in which cerebral vascular plugging was present had non-cerebral deaths.

Sequestering parasitized red cells are often an obvious histological feature, so some researchers [5,8,302] continue enthusiasm for vaso-occlusion being the prime mover of falciparum disease, with the cytokines of systemic inflammation reserved, at best, for justifying what are seen as peripheral phenomena such as fever and nausea. Indeed, the notion of the vomiting and nausea of severe malaria being best explained by intestinal vacular sequestration is still put forward [174], many years after TNF, the major cytokine increased in the circulation in severe malaria, was shown to produce these changes when injected into volunteers [150]. To be credible as the major initiating cause of malarial coma, any proposed mechanism has to reconciled with the low incidence of residual neurological deficiency in cerebral malaria survivors compared to that seen after the archetypal cerebral vaso-occlusive state, stroke, in which even a short period of coma predicts severe functional loss [106]. It also needs to explain how malaria-tolerant individuals routinely avoid coma (or indeed illness), since sequestration of infected red cells is a normal part of the biology of the parasite, and the parasite density in tolerant children can be quite high [303]. Clearly, non-vasoocclusive mechanisms for malarial coma can accomodate these two commonly reported phenomena. The recent emphasis, when considering the pathogenesis of severe illness, on total parasite load (implying a corresponding level of malaria toxin-induced mediators of host origin), rather than the degree of sequestration they cause [174], is a welcome development towards acceptance of the cytokine concept.

Human babesiosis is a haemoprotozoan tick-borne disease often associated with symptoms that are similar to falciparum malaria [304,305]. Species that cause human infections include *Babesia microti*, the mouse form of which is functionally allied with several mouse malaria species, in that they cross-protect and cause similar pathology [306]. Complications in human *B. microti *infection can include altered mental status, adult respiratory distress syndrome, renal insufficiency, disseminated intravascular coagulation (DIC), gastrointestinal bleeding and multi-organ failure [304,305]. These changes have been assumed, in parallel with the tradition for human malaria, to be caused by vascular occlusion. Since *B. microti *does not sequester in mice, a recent opportunity was taken to see if it behaved differently in tissues from a patient infected with this parasite. Sequestration was not observed [307]. The implications, from this single case study (a splenectomised individual, but the pathology can be equally complex in intact and asplenic patients [308,309]), implies that another mechanism, such as high cytokines [310], must be the cause of the falciparum-like pathology of this disease. It is also appropriate to note here that selection for degree of sequestration of *Babesia bovis*, widely regarded as a parallel for the vaso-occlusive model of falciparum malaria, did not produce parasites that generated more virulent infections in cattle [311].

As English and co-workers have noted, the low mortality when coma occurs in the absence of anaemia or markers of metabolic acidosis indicates that a localised cause of coma, such as cerebral vaso-occlusion, is very unlikely to be a sole cause of mortality without a systemic inflammatory input, and that coma in acidotic children might well have a different mechanism than in those with brain lesions only [312]. Nevertheless, loyality to the vaso-occlusion explanation of malarial coma and death can be strong, leading, in one instance, to several interpretations of the same autopsy series [8,71,313]. One version [8] discounts evidence of systemic inflammation [71] and argues that cerebral malaria should be redefined as an autopsy-based, cerebral sequestration-defined entity rather than, as is usual, a clinical syndrome with the capacity to encompass a range of mechanisms, local or systemic, thought able to generate the same clinical endpoint [300,314]. The need to treat ill individuals and conduct clinical research in circumstances when patients survive, or autopsies are impractical, ensures that the present standard clinical definition is not likely to be displaced. Indeed in a recent publication on microparticles [140] the laboratory seeking to give cerebral malaria an autopsy-based definition [8] itself reverted to the standard definition, of necessity. Final decisions on what can be learnt from the only large African paediatric autopsy study to date await publication of the autopsy reports from this study, and correlation with the information already in the literature on these cases [71,252].

If cerebral sequestration proves not to become prominent until soon before death, might it, though present at autopsy, have been incidental to the fatal outcome? There is now increasing agreement [69,314,315] with insights, now decades old [19,316], that the coma of falciparum malaria can often be a cytokine-induced metabolic encephalopathy. Thus, a consensus appears to be developing that, despite how technically impressive knowledge of the mechanism of adhesion is becoming [317], the rapid and usually complete resolution of cerebral symptoms in most (presumably even those with cerebral sequestration) who recover is inconsistent with a primary role for mechanical microcirculatory obstruction. This adds to the evidence that it seems ill-advised to regard fatal coma in malaria-infected African children as having been caused by malaria only when sequestration is present in brain sections [8].

Functional evidence, in terms of reduced blood flow, has been sought for cerebral vascular occlusion being important in the pathogenesis of disease during falciparum malaria, both in Thai adults [318] and African children [319], but not found. Indeed, increase in cerebral venous pO_2 _was noted at the time [318] to be consistent with an inability to utilise the oxygen delivered, a cytokine-dependent concept discussed above. Another approach has capitalized on variation in the degree to which different strains of parasite cause adhesion of the red cell they inhabit. One study found no association between disease severity and adherence to CD36, and significance for ICAM-1 when anaemic cases are discarded [320], while another reported a significant inverse correlation of illness severity with both binding sites [321]. In contrast to the traditional story, this implies that sequestration could be associated with a less pathogenic infection. The tools to test anti-PfEPM1 in this context have been available for several years, and demonstrated to work in the human/severe combined immunodeficiency (SCID) mouse chimeric model [322], but to our knowledge there are no reports of them having been tested clinically.

The outcome of the recent study in African children referred to above [71] provides a very useful window on "pure" falciparum malaria compared to cases in which death may have occurred sooner because of another contributing factor. One third of the 32 fatal clinically-defined cerebral malaria cases examined histologically had little or nothing to see in the brain by way of sequestered parasites, monocyte clusters, microhaemorrhages, or iNOS or HO-1 staining that would have indicated the advent of local vascular inflammatory change at the time of death. Some had high parasitaemias [71] and strong gut vessel sequestration of parasitised red cells (unpublished). Yet this third was clinically diagnosed as cerebral malaria on the same criteria as were the rest, presumably providing a modern day example of the cases described by Kean and Smith [301]. Two of this group had severe anaemia, and others, on autopsy, proved to have non-malarial changes, such as bacterial pneumonia or patchy hepatic necrosis. These changes were judged to have contributed to the fatal outcome, leading to an earlier autopsy, in malaria terms, than would have happened in the absence of this joint cause of death. As discussed [107], this group, one third of the cases, gives an useful insight into what may have been happening within the cerebral vasculature of the rest of these patients, the "pure" malaria cases, after they had become comatose, but were still some time away from death, when cerebral sequestration, monocytes, platelets and microhaemorrhges were present [71].

Intriguingly, these comatose malaria cases with no appreciable change in their brains nevertheless showed appreciable systemic evidence of inflammation, as demonstrated by the marked evidence of iNOS, MIF [71] and HO-1 [252] expression elsewhere. These systemic changes were shared with the comatose sepsis cases in the series – indeed there was nothing to tell them apart immunohistochemically, and the depth of coma was of the same order in both groups. The outcome in most of these cases was likely to have been determined by the acute downstream effects of inflammatory cytokines, including energy depletion and metabolic acidosis, although data that could have provided this degree of detail was unavailable from these individual cases. The rest of the cases, with a range of degrees of sequestration and evidence of systemic inflammation, are discussed in detail in a recent review [21].

From the principles that form the stroke literature, individuals with appreciable cerebral sequestration are likely to be over-represented in fatal cases. Likewise, comatose patients who do not develop local brain changes, including overt sequestration, might well have a higher chance of survival, and are thus under-represented in the fatal comatose cases from which are drawn the histology samples that are the basis of the sequestration theory of cerebral malaria. Recent work on mouse cerebral malaria, caused by *Plasmodium berghei *ANKA, has used new experimental tools to address this question, and in a detailed and convincing study goes against conventional wisdom, disassociating cerebral pathology from sequestration [152]. This research warrants being given due weight by those advocating the relevance of this model to the primary lesion in human cerebral malaria. The next section of this review provides examples of cytokine-induced metabolic encephalopathies that cast light on the coma of falciparum malaria.

#### Systemic inflammation, no parasite sequestration, yet still encephalopathy

To understand cerebral functional impairment, including coma, in clinically defined cases of falciparum cerebral malaria that show minimal if any sequestration on brain sections [18,71], it warrants considering which other infectious systemic diseases can cause a focal encephalopathy. In 2002, Kunze provided an excellent overview [323] in which he discussed metabolic encephalopathies in general under hypoxic, ischaemic, systemic (including infectious) and toxic groupings. It seems clear that changes occurring in falciparum malarial encephalopathy qualify it for inclusion in several of these categories without the presence of the sequestered parasites. This 2002 review provides adequate material from which to argue that an intense inflammatory response, in the sense of a strong systemic pro-inflammatory imbalance of cytokines, as distinct from the traditional cellular influx, can cause a cerebral syndrome essentially the same as that observed in severe falciparum malaria.

##### Cerebral malaria caused by *Plasmodium vivax*

Probably the most immediately relevant of these encephalopathies to a discussion on falciparum malaria (for it is just as validly termed cerebral malaria) is the reduced consciousness sometimes seen in severe vivax malaria, caused by a parasite accepted not to sequester. For many years such reports were dismissed as undiagnosed double infections (vivax plus falciparum), but evidence from geographical areas where falciparum malaria was not present, plus new specific diagnostic techniques, have led to an acceptance of the concept. For instance, in 2002 Beg collected the records of 41 such cases [136], and a more recent study described 11 such patients [137]. They exhibited cerebral symptoms, renal failure, circulatory collapse, severe anaemia, haemoglobinuria, abnormal bleeding, acute respiratory distress syndrome and jaundice, all of which are commonly associated with falciparum malaria. Vivax malaria can demonstrate a strong systemic inflammatory response [324], but this was not examined in these cases. Anstey and co-workers have compared a range of pulmonary functions in vivax and falciparum infections, and concluded that a common underlying inflammatory mechanism was probably acting [325].

##### Sepsis encephalopathy

As in falciparum malaria, mental confusion and obtundation can be early signs of the sepsis syndrome, and altered mental status is associated with a higher mortality [326]. The clinical features of sepsis encephalopathy vary from mild confusion to coma, one study of 69 sepsis cases finding 32 with severe functional changes [323]. This study showed that in sepsis the brain fails in parallel with other organs, and that the severity of the systemic illness, rather than the encephalopathy *per se*, accounts for the outcome in individual patients, although the brain proves to be a sensitive indicator of severe generalised septic illness. More recently, Morita and co-workers [327], studying 27 acute sepsis encephalopathy cases, including some asssociated with influenza (see next section), found inflammatory cytokines to be higher in the serum than in the cerebrospinal fluid. They also reported that high serum inflammatory cytokine levels at the initial stage of illness, and the good correlation of those levels with outcome, suggest that systemic inflammation has a significant role in the blood-brain barrier permeability, general vascular leakage and multiple organ dysfunctions in patients with acute encephalopathy. Evidence of causality has been observed in an animal model in which prior administration of a neutralizing antibody to TNF prevented the sepsis encephalopathy, generated through experimental pancreatitis, a standard functional example of sepsis [328].

##### Influenza encephalopathy

Probably the best documented encephalopathy which closely parallels the changes that may occur in falciparum malaria is the form sometimes seen in severe influenza. It is most frequently reported in children in Japan and other Asian countries, and provides a close functional match indeed. As in malaria, the infectious agent is not neuroinvasive [329]. Seizures and coma occur after high grade fever [330], commonly accompanied by thrombocytopaenia [330], and metabolic acidosis and hyperlactataemia in severe cases (T. Ichiyama, pers. comm.). TNF, IL-6, sTNFRI, and soluble E-selectin are increased in serum and CSF [331,332], and serum nitrite/nitrate levels are increased [333]. Vascular endothelial cells are damaged [135] and multiple organ failure and neurological sequelae occur [334], as does brain oedema associated with damage to the blood-brain barrier [335]. More detailed examination of brain has revealed apoptosis of neurons and glial cells [335], as well as histological evidence of active caspase-3 [335], and caspase-cleaved PARP [335]. As summarized in Table 2, these changes are all demonstrable in the encephalopathy of falciparum malaria, implying that in this disease sequestering parasitized erythrocytes may accompany, rather than cause, them. The concept of cytokine-induced mitochondrial respiratory failure, as discussed above, is a topic of interest in influenza encephalopathy research [336]. It is noteworthy that circulating levels of cytochrome c, released from damaged mitochondria, are increased in both sepsis [337] and influenza encephalopathy [332], in which it has proved to have better prognostic predictive value than do inflammatory cytokines [338]. This provides a strong case for it also being tested in severe malaria.

It is feasible, but to our knowledge not yet investigated, that increased inflammatory cytokines might cause some degree of poor brain microcirculation in influenza encephalopathy, as well as some local enhancement of inflammation, through inducing adhesion of platelets and monocytes. Were this so, it would be reasonable to regard such changes as pathology-enhancing accessories after the event was under way rather than prime instigators of it. In summary, influenza encephalopathy demonstrates that a syndrome inseparable from cerebral malaria can occur without invoking adherent parasitized red cells. The evidence suggests that the CM(A) group of paediatric malaria cases [71], and well as the earlier Kean [301] and Maegraith [18] examples, are conceptually the same as influenza encephalopathy.

##### Hyperphenylalanaemia and IDO induction in systemic infectious diseases

Phenylalanine is an aromatic amino acid required, in controlled concentrations, for the normal synthesis of various monoamines essential for brain function. Several types of genetic defects of phenylalanine hydroxylase or tetrahydrobiopterin metabolism can elevate its plasma levels to pathological levels. If not corrected by a low phenylalanine diet from an early age, chronic excess of this amino acid can cause an encephalopathy associated with seizures, and in time retards brain development and causes various functional deficits [339]. Transient hyperphenylalaninaemia can be induced by endotoxin [340] or TNF [341], so not surprisingly is a component of the various systemic inflammatory states. For example phenylalanine is reported to be increased (20.9-fold in CSF and 7.5-fold in plasma) in septic encephalopathy [342]. In another study the increase correlated with APACHE II score [343]. The same increase in severe falciparum malaria, particularly in comatose children [344,345], is entirely consistent with this condition also being another systemic inflammatory state, a main theme of this review. Therefore, as in sepsis encephalopathy, a reasonable case can be made for transient hyperphenylalaninaemia to be an additional inflammation-associated mechanism for encephalopathy in malaria, independent of vascular occlusion. So too, as reviewed recently elsewhere [315], does the induction, by inflammatory cytokines, of indoleamine 2,3-dioxygenase (IDO), a rate-limiting enzyme in the L-tryptophan-kynurenine pathway, which converts L-tryptophan, an essential amino acid, to N-formylkynurenine. The observations that this also occurs in sepsis [346] and trauma [347], also strengthen our original case [348] for organ failure in malaria, sepsis and trauma having the same pathophysiology.

#### Seizures in encephalopathies

As noted above, hyperphenylalanaemia warrants serious consideration as a contributor to the seizures observed in septic encephalopathy and cerebral malaria. So too does zinc chelation by S100 proteins. Our 2003 studies on tissue sections from East African children [71,252] included a number of cases of severe sepsis encephalopathy, without malaria parasites but with bacterial pathogens (eg *Escherichia coli *and *Salmonella enteritidis*) present. Certain tissues exhibited immunohistochemical evidence (iNOS, MIF and HO-1) of intense systemic inflammation. *S. enteritidis *is a pathogen known to alter brain function, causing a diffuse encephalopathy, including seizures as part of a systemic syndrome [349]. Current ideas on the origin of septic encephalopathy, including blood-brain barrier opening and S100B as a possible marker for the degree of pathology, have been discussed recently [350]. S100B has also been reported to be increased in cerebral malaria in Vietnamese adults, where it was associated with seizures [351].

**Figure 5:**
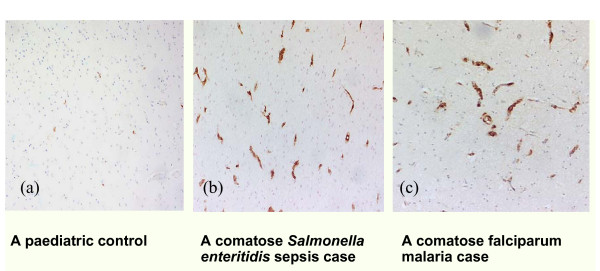
Immunohistochemical staining of cerebrum of (a) African paediatric control (MP41), (b) *Salmonella enteritidis *(MP12) and (c) *P. falciparum* (MP6) to detect S100A12. Techniques (DAB, haematoxylin) as in reference 71. Antibody courtesy of Dr Carolyn Geczy. See Table 1 of ref. 71 for case details. Unpublished data.

Serum and CSF Zn levels decrease during infectious diseases, and this decrease is more significant in patients with febrile convulsions [352]. Both S100B and other members of the S100 protein family, including S100A12, are strong chelators of zinc [353,354]. S100A12 is an IL-1 and TNF-inducible mediator found to be increased in severe malaria serum (M. Griffith, C. Geczy and I. Clark, submitted) and detected in monocytes in cerebral vessels in falciparum malaria and *Salmonella enteritidis *sepsis (Figure 5). Cerebral zinc homeostasis is essential for normal brain function [355] and, of relevance to seizures, zinc induces the spontaneous depolarising synaptic potentials that normally dampen synaptic transmission. These potentials can be inhibited by zinc chelation [356]. These authors note the implications of this result for understanding seizures in young children with acute dietary zinc deficiency. The concept warrants exploration in the seizures in the encephalopathies of sepsis, influenza and malaria in young children, since S100B and cytokine-induced S100 proteins can be expected to act like these synthetic zinc chelators [356] and generate local areas of functional zinc deficiency.

### Inflammatory cytokines offer insights into differences between childhood and adult malaria

There are real differences in the nature of the disease in children and adults. For example, severe anaemia is much more common for a given parasite density in young children than in older children and adults [300], and seizures follow a similar pattern [357]. In contrast, cerebral malaria is less common in the very young, and the pulmonary and renal complications seen in adults are very uncommon in children [300]. Moreover, children can experience a type of clear lung respiratory distress not reported in adults [358], implying a more severe metabolic acidosis driven by the need to blow off excess CO_2 _and restore normal pH. This is consistent with hypoglycaemia (from high glucose consumption feeding the anaerobic state that is causing proton accumulation) being a characteristic of childhood malaria more than of adult cases.

The cytokine theory of disease provides directions that could lead to an understanding, currently lacking, for these age differences. For instance, generation of nitric oxide, which has been instrumental in the Na^+^/K^+^-ATPase inhibition thought to cause poor red cell deformability, and thus shorten erythrocyte life span, is higher in younger children [359,360]. Additional nitric oxide generated by a systemic inflammatory response would add to this higher base level, reducing red cell deformability correspondingly more in the very young. This could be tested. Likewise, pulmonary and renal organ failure being common in severely affected adults, but virtually absent in children [300], has an exact parallel in the age differences for the inflammatory response that follows trauma [361]. This has been explained in terms of the different balance of inflammatory cytokines generated from normal peritoneal cells collected from children vs adults [362,363]. There seems no reason why these data, derived from normal human cells, should not be equally applicable to any human systemic inflammatory state, including malaria. This enhanced anti-inflammatory response of young children can also be expected to minimize the proposed focussing of sequestration during severe illness in this age group, contributing to their low incidence of cerebral malaria [300].

## Conclusion

In broad terms, the essential mechanism of death in falciparum malaria disease is agreed by many researchers: a functional tissue hypoxia that forces an unsustainable dependence on anaerobic metabolism. An unresolved key question is whether the tissue hypoxia arises (a) because insufficient oxygen reaches the mitochondria through either vascular occlusion from sequestered parasitized red cells acting alone, or in combination with anaemia or (b) because excessive release of inflammatory cytokines, induced by malarial toxin(s), render mitochondria unable to use oxygen to generate energy from oxidative phosphorylation. Although wearing contemporary clothes, this is the same dichotomy that was present a hundred years ago. Current basic literature suggests that inflammatory cytokines are very much the dominant partner, having amongst their powers the capacity to shut down bone marrow, make red cells prematurely poorly deformable, and channel sequestration towards certain sites, dictated by inate local thrombomodulin concentration (Figure 1). Thus cytokines and poor oxygen delivery should not be viewed as alternative theories of malarial disease pathophysiology. Instead, the latter is one of the consequences of the former.
